# Separation of Light and Heavy Rare Earth Elements via Electrospun Supported Liquid Membrane

**DOI:** 10.3390/polym18141742

**Published:** 2026-07-16

**Authors:** Shafiq Mohd Hizam, Nur Syakinah Abd Halim, Nik Nurul Aiman Zulaika Nik Hanafi, Aida Syafiqah Abdul Manaf, Mohd Dzul Hakim Wirzal, Yew Mei Quen, Santosh Mishra, Chrisminder Dain

**Affiliations:** 1 Chemical Engineering Department, Universiti Teknologi PETRONAS, Bandar Seri Iskandar 32610, Perak, Malaysia; shafiq.hizam@utp.edu.my (S.M.H.); syakinah.halim@utp.edu.my (N.S.A.H.); zulaikha.hanafi@utp.edu.my (N.N.A.Z.N.H.); syafiqah.manaf@utp.edu.my (A.S.A.M.); 2 PETRONAS Research Sdn Bhd, Bandar Baru Bangi 43000, Selangor, Malaysia; yew.meiquen@petronas.com (Y.M.Q.); santosh.mishra@petronas.com (S.M.); hariz_chris@petronas.com (C.D.)

**Keywords:** supported liquid membrane (SLM), electrospun membrane, selective separation, light and heavy REEs (LREEs/HREEs)

## Abstract

Rare earth elements (REEs) are predominantly separated by using mixer–settler solvent extraction technology. While this method has been utilized in the industry, it does have a few setbacks, such as requirement of large module footprint to compensate for inefficient separation. Likewise, researchers have turned to membrane separation technology to address these challenges faced by mixer–settler solvent extraction. Supported liquid membranes (SLMs) are a viable alternative in membrane separation technology as they exhibit similar conceptual designs to that of solvent extraction, albeit requiring smaller amounts of organic carrier and lower module footprint compared to mixer settler solvent extraction. In this research, the application of an electrospun membrane was explored using SLMs for the separation between light REEs (LREEs) and heavy REEs (HREEs). To do this, the study initially employs the usage of neodymium (Nd) and dysprosium (Dy) as the baseline for LREEs and HREEs, and later using real REE leachate for the separation between LREEs and HREEs. This study also investigates the effects of different pH of feed solution, organic carrier loading, and stripping concentrations. Overall, to achieve higher selectivity of Nd/Dy, a higher pH of feed solution of 5, a low loading of organic carrier at 10 wt%, and a low stripping concentration of 1 M H_2_SO_4_ was recommended. For the separation of LREEs and HREEs, a separation factor of 2.38 was achieved with a recovery of 10.43% of LREEs in under 60 min by using a small-scale membrane of 4 cm^2^ effective area. Long term operation indicates a stable LREE/HREE separation performance using the optimized condition.

## 1. Introduction

Rare earth elements (REEs) are a group of 17 lanthanide metals [1]. Despite being called “rare,” these elements are quite abundant in the Earth’s crust, but they are usually found in dispersed forms rather than in large, concentrated deposits. Because of the relatively close atomic numbers between adjacent REEs, they exhibit similar chemical and physical properties. In recent years, REEs have gained increasing attention due to the rapid growth of renewable energy technologies and geopolitical factors. Among these elements, neodymium (Nd) and dysprosium (Dy) are particularly important, as they are widely used in advanced energy applications such as batteries and permanent magnets [2].

One of the primary challenges in separating rare earth elements (REEs) is their chemical similarity. Since all REEs exhibit comparable electronic configurations and oxidation states (typically +3), their chemical behavior is nearly identical. Additionally, their ionic radii differ only slightly, making conventional separation techniques, such as solvent extraction, highly complex and inefficient [3]. These similarities necessitate multiple, energy-intensive separation steps to achieve high-purity individual REEs [4].

Several conventional methods were implemented for extraction and separation of REEs, and one of them is solvent extraction. Solvent extraction, also known as liquid–liquid extraction, is a widely employed technique for separating components based on their differential solubilities in two immiscible liquids, typically an aqueous phase and an organic solvent [5,6]. However, solvent extraction has a few limitations, such as high organic carrier consumption [7,8], which leads to the increase of operational costs besides the formation of emulsions which complicate the phase separation, hence reducing the separation efficiency [9].

**Figure 1:**
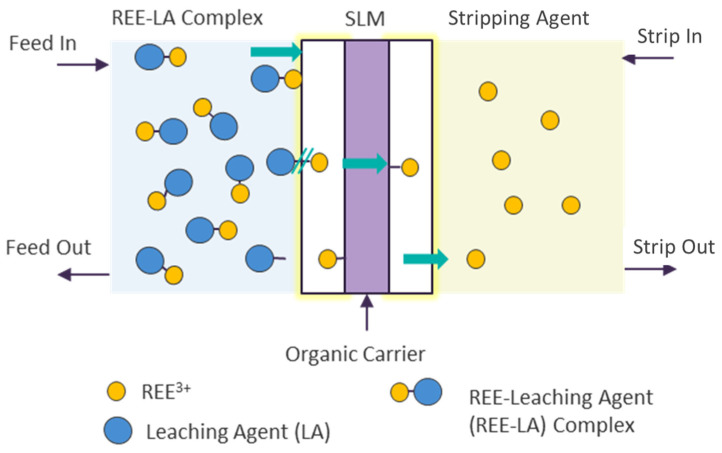
Conceptual process flow diagram of SLM and its principal mechanism in transporting REE.

To address these challenges, supported liquid membranes (SLMs) have emerged as a promising alternative for REE separation. SLMs consist of a liquid extractant, or organic carrier, immobilized within the pores of a solid support, effectively combining the principles of solvent extraction and membrane separation. Figure 1 shows the conceptual process flow diagram for a SLM. In contrast to solvent extraction, SLMs require smaller amounts of extractants compared to traditional solvent extraction, reducing operational costs and chemical waste generation [10,11]. Several studies have been conducted to investigate the efficiency of SLMs system in REE separation. Kim et al., (2016) have demonstrated the use of polypropylene hollow fiber membrane for the recovery of Nd^3+^ and Dy^3+^ using tetraoctyl digylcol amide (TODGA) and Cyanex 923 as the organic carrier [12]. The result shows remarkable recovery of REEs (>80%) from a NdFeB permanent magnet. A study conducted by Tehrani and Kelishami, (2019) [13] highlights the use of SiO_2_ nanoparticles in PTFE membrane with Aliquat-336 and kerosene as the organic carrier for the separation of Gd^3+^ and Nd^3+^. It was shown that an improvement of 28.3% and 49.5% permeability coefficient for Gd^3+^ and Nd^3+^ extraction, respectively, using hydrophobic nanoparticle SiO2. This may also be due to an increase in active area with nanoparticles. Another study by Middleton et al., (2023) focuses on the effect of feedstock variables (Al, Fe, pH) on Nd and Er recovery flux [14]. It was found that REE recovery increases with higher pH. The permeability coefficients of Nd and Er were most sensitive to the feedstock concentration of Fe^3+^ relative to Al^3+^ and Fe^2+^.

Typically, SLMs use a phase inversion fabrication technique or commercialized PVDF membrane as the porous membrane support for the REE separation [15,16]. Phase inversion is a widely employed method for fabricating polymeric membranes, including those used as supports in SLM systems. This process involves transforming a homogeneous polymer solution from a liquid to a solid state through controlled phase separation. The primary techniques for inducing phase inversion includes thermally induced phase separation (TIPS), nonsolvent-induced phase separation (NIPS), vapor-induced phase separation (VIPS), and exchange of solvent with nonsolvent [17,18]. Nevertheless, phase inversion membranes typically exhibit a denser structure with limited porosity and fewer accessible pore pathways, which can restrict the diffusion and immobilization of carrier molecules, in this case impregnation of di(2-ethylhexyl)phosphoric acid, DEPHA.

Henceforth, electrospinning shines its way in producing membranes with high porosity to improve membrane efficiency. Electrospun membranes are known to have high porosity, huge surface to volume ratio, and tunable pore size [19,20,21]. The application of electrospun membranes is highly common in wastewater treatment, including heavy metals removal [22]. A study conducted by Sahoo et al. focuses on the development of electrospun magnetic polyacrylonitrile–graphene oxide (PAN-GO) hybrid nanofibers [23]. These nanofibers are synthesized to enhance the removal of hexavalent chromium (Cr(VI)) from aqueous solutions. The results show that the PAN-GO-Fe_3_O_4_ electrospun nanofiber membrane has a higher surface area (90.62 mg/g) with a maximum adsorption capacity of 124.34 mg/g.

Due to the excellent capabilities of electrospun membrane in heavy metals removal, this study incorporates an electrospun membrane with bis(2-ethylhexyl) phosphate (DEPHA) as the organic carrier with sulfuric acid as the stripping agent were used in a supported liquid membrane (SLM) system to evaluate the separation performance between neodymium (Nd) and dysprosium (Dy). The carrier immobilization of DEPHA and kerosene within the membrane pores is via capillary forces and interactions with the PVDF matrix [24]. In this study, the PVDF electrospun membrane acts as a supported liquid membrane, where the DEPHA/kerosene phase is immobilized within the porous structure of the PVDF fibers and serves as the carrier phase for REE transport. The open-pore structure of the electrospun membrane provides continuous pathways for the liquid phase to be held within the pores. The immobilization is mainly assisted by the capillary force, where the strong affinity causes kerosene to be drawn into the pores by capillary action. In addition, the hydrophobic nature of PVDF promotes better affinity with the organic phase, which further enhances the stability of the DEPHA/kerosene within the membrane. This study also investigates the factors that affects the separation performance of SLMs, including the pH of the feed solution, organic carrier concentration, and stripping agent concentration. The goal of separating Nd and Dy was to evaluate the separation efficiency between light REEs (LREEs) and heavy REEs (HREEs) by using SLMs. Herein, it is worth noting that Nd represents the LREE group and Dy represents HREE group. Afterwards, through a one-time factor optimization approach based on the aforementioned factors, the optimized parameters will then be validated using real REE leachate or pregnant leaching solution (PLS) samples.

## 2. Methodology

### 2.1. Materials

Neodymium (III) nitrate hexahydrate (NdNO_3_, 99.9% trace metal basis, Sigma Aldrich, St. Louis, MO, USA) and dysprosium (III) nitrate hexahydrate (DyNO_3_, 99.9% trace metal basis, Sigma Aldrich, St. Louis, MO, USA) were used for synthesis of feed solution. Sulfuric acid (H_2_SO_4_, 95–97% EMSURE, Merck, Rahway, NJ, USA) was used as a stripping solution. Polyvinylidene fluoride (PVDF) pellets (MW 530,000, Merck, Rahway, NJ, USA), N,N-Dimethylacetamide (DMAc, 99%, Reagent Plus, Merck, Rahway, NJ, USA) and lab grade acetone (99.5%, Merck, Rahway, NJ, USA) were used for the synthesis of polymer solution. Kerosene (low odor, Merck, Rahway, NJ, USA) and bis(2-ethylhexyl) phosphate (DEPHA, 95%, Thermo Fisher Scientific, Waltham, MA, USA) were used as the organic carrier. A pregnant leaching solution (PLS) was prepared under the support of PETRONAS Research Sdn Bhd (PRSB, Bangi, Selangor, Malaysia), which consists of REE ions leachate. For the sole purpose of this study, only REEs were focused on for analysis from the PLS. Potassium hydroxide (KOH, 90% reagent grade, Merck, Rahway, NJ, USA) and nitric acid (HNO_3_, 65% reag. Ph. Eur., Merck, Rahway, NJ, USA) were used to adjust the pH of the feed solution.

### 2.2. Membrane Preparation

PVDF solution was first prepared by dissolving PVDF pellets (25 wt%) in a mixture of dimethylacetamide (DMAc) and acetone. The solution was stirred overnight at 60 °C until a homogeneous solution was formed. Next, membrane fabrication was conducted via electrospinning. Upon fabrication, the PVDF solution was filled inside a 5.0 mL syringe. The parameters for electrospinning are summarized in Table 1.

Once the electrospun membrane was completely formed, the membrane was cut into smaller pieces (4 cm × 4 cm). The membrane pieces were immersed and sonicated for 60 min in different loading of organic carrier solution (mixture of DEPHA and kerosene). Here, the organic carrier used was a mixture at a specific loading of DEPHA at 10 wt%, 20 wt%, and 30 wt%, with the remaining percentage being kerosene, respectively.

### 2.3. Membrane Characterization

The membrane was characterized based on functional group, surface morphology, and elemental analysis. To analyze the surface morphology/elements, Field Emission Scanning Electron Microscope–Energy Dispersive Spectroscopy (FESEM–EDS, Model: VPFESEM, Zeiss Supra55 VP, Oberkochen, Germany) was used. Fourier Transform Infrared Spectroscopy (FTIR, Model: FTIR Frontier, Perkin Elmer, Shelton, CT, USA) was used to identify the chemical bonds and the presence of functional groups.

### 2.4. Feed and Stripping Solution Preparation

A stock solution of NdNO_3_ and DyNO_3_ was prepared by dissolving 50 mg of the respective salts with deionized (DI) water in a 1 L volumetric flask. To prepare the feed solution, 75 mL of each NdNO_3_ and DyNO_3_ solution were mixed in a separate container. The feed solution was then measured using Inductively Coupled Plasma Mass Spectrometry, ICP-MS (Model: Agilent, Santa Clara, CA, USA) to determine initial concentrations of both Nd and Dy. Next, the stripping solution was prepared by diluting H_2_SO_4_ into 1 M, 3 M, and 5 M concentration with DI water. For each test, about 150 mL of stripping solution was used.

### 2.5. Separation Between Nd and Dy

**Figure 2:**
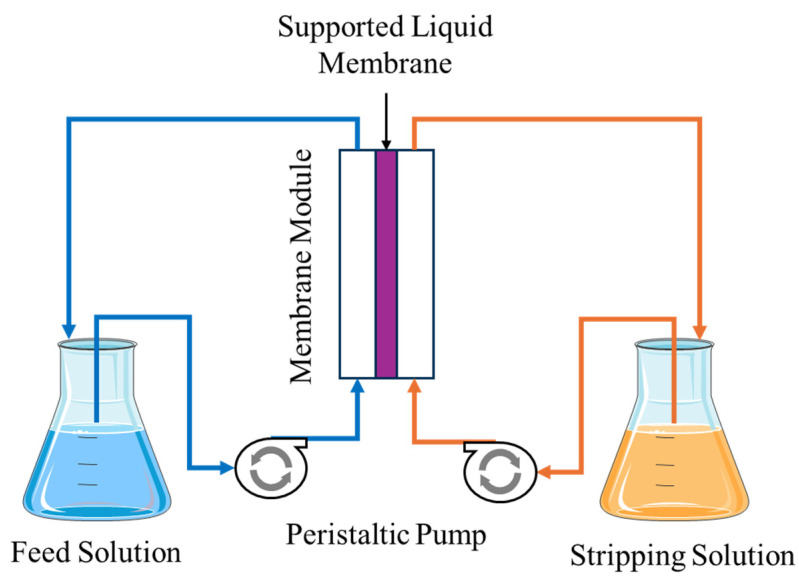
Setup for the separation of Nd and Dy experiment.

The feed and stripping solution were prepared 150 mL each in a separate container as outlined in Section 2.4. The immersed electrospun membrane was placed on a membrane module with an effective area of 4 cm^2^. Prior to the start of the experiment, the initial feed solution was collected and sent to ICP-MS to determine the initial concentration of Nd and Dy. Next, the experiment begun by circulating both feed and stripping solution through a peristaltic pump at a flowrate of 90 mL/min for 60 min. At each 10 min interval, a sample from the feed and stripping solution was collected and sent to ICP-MS for analysis. Each result shown in Section 3.2 is a triplicate result shown as an average. Figure 2 shows the setup for the separation of Nd and Dy experiment.

To determine the distribution coefficient of Nd and Dy, one must determine the concentration of the respective species at the stripping solution and the feed solution [15]. The formula for distribution coefficient is outlined in Equation (1):

(1) $$D_{A}=\frac{C_{A,strip}}{C_{A,feed}}$$

where *D_A_* denotes the distribution coefficient of species A, *C_A,strip_* refers to the concentration (ppm) of species A in the stripping solution, and *C_A,feed_* indicates the concentration (ppm) of species A in the feed solution. Note that the concerned species, A in this work applies to both Nd and Dy.

To investigate the separation factor between species, one can determine through the ratio between distribution coefficient between two species A and B as describe elsewhere [25,26]. The formula for separation factor is shown in Equation (2):

(2) $$\beta_{A/B}=\frac{D_{A}}{D_{B}}=[\frac{C_{A,\;strip}}{C_{A,\;feed}}]/[\frac{C_{B,\;strip}}{C_{B,\;feed}}]\;$$

where *β_A/B_* represents the separation factor between species A and B, with emphasis on species A over B, *D_A_* signifies the distribution coefficient of species A, and *D_B_* stands for distribution coefficient of species B. Note that the separation factor can be interchangeable between species A and B, depending on viewpoint. However, this work emphasizes on separation factor of Nd over Dy (*β_Nd/Dy_*).

Lastly, to determine the recovery of Nd and Dy, one can determine through Equation (3):

(3) $$Recovery\;\left(\%\right)=\;\frac{C_{A,strip}-C_{A,feed}}{C_{A,feed}}\;\times 100\%$$

where *C_A,strip_* refers to concentration of species A in the stripping solution and *C_A,feed_* denotes concentration of species A in the feed solution. Note that species A applies to both Nd and Dy.

In this research, the experiment was conducted to study on different factors that will affect the separation performance of the SLM. These factors include pH of the feed solution (pH 0.7, 1.85 and 5), organic carrier loading (DEPHA loading of 10 wt%, 20 wt%, and 30 wt%), and stripping concentration (H_2_SO_4_ concentration of 1 M, 3 M, and 5 M). A summary of the parametric study is outlined in Table 2. Hereafter, the aforementioned factors underwent one factor approach optimization and were tested using real PLS samples prepared under the funding support of our research partner, PETRONAS Research Sdn Bhd (PRSB). To assess the behavior of LREE and HREE under long term operation, the experiment was subjected to 5 h continuous operation using the same setup as shown in Figure 2, with interval sampling of both feed and stripping solution.

## 3. Results and Discussion

### 3.1. Membrane Characterization

**Figure 3:**
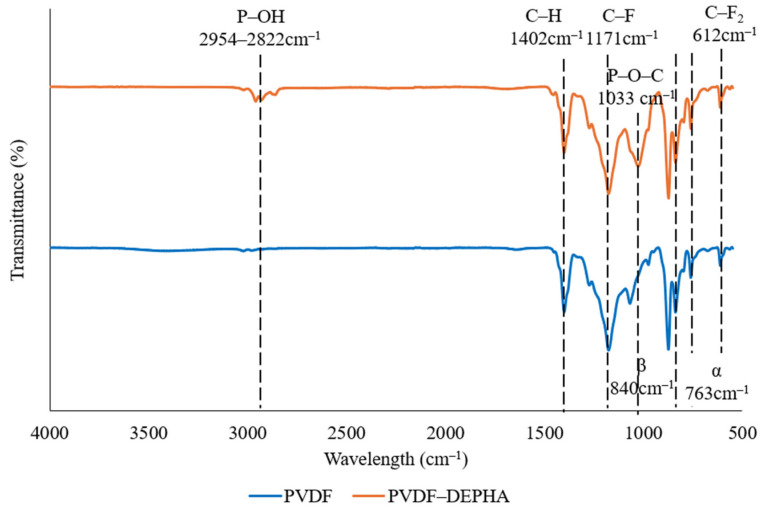
FTIR spectrum of PVDF and impregnated PVDF–DEPHA.

For this study, the PVDF membranes were fabricated through electrospinning, as outlined in Table 1. Among the characterization of the fabricated membranes are FTIR and FESEM-EDS. Figure 3 shows the FTIR spectrum of neat PVDF and impregnated PVDF with DEPHA/kerosene. The neat PVDF membrane contains a certain discrete spectrum in which it corresponds to its molecular structure. Of a particular functional group, PVDF consists of C-H and C-F bonding. The C-H stretching vibrations appear at the peak of 1402 cm^−1^ while a strong absorption band appears in the 1171 cm^−1^ region due to CF stretching vibrations. Another peak observed was at 612 cm^−1^, where it indicates C-F_2_ bonding. The α and β phase of the PVDF matrix appear at 763 cm^−1^ and 840 cm^−1^, respectively. These peaks were similar to the work done by Sun, et al. (2022) [27].

In the case of PVDF-DEPHA, the bands appeared that mark the absorption spectrum for phosphate-based compounds compared to neat PVDF. A strong band of P-O-C stretching vibrations appears in the region of 1033 cm^−1^, which supports the presence of phosphate esters. There was also a P-OH bonding observed between 2954–2822 cm^−1^ region, though slightly deviated from the work done by Parhi, et al. (2018) [28]. The work done by Parhi, et al. (2018) also revealed the spectra for kerosene, which has a similar spectra to that of DEPHA, particularly in the 2750–2550 cm^−1^ region [28]. From this, it can be concluded that DEPHA/kerosene has successfully been impregnated within the membrane pores.

**Figure 4:**
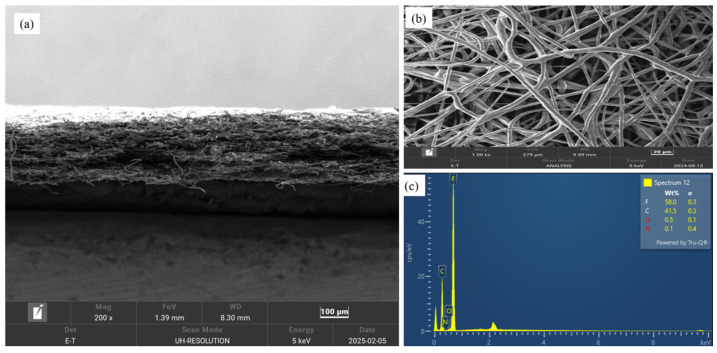
(**a**) FESEM image of membrane cross-section at 200× magnification, (**b**) FESEM image at 1000× magnification, and (**c**) EDS result for neat PVDF membrane.

FESEM-EDS analysis was conducted to determine the fiber diameter and to evaluate the chemical composition of the membrane before and after incorporation of DEPHA/kerosene. Figure 4a shows the FESEM image of the electrospun membrane at its cross-section at 200× magnification. The FESEM image reveals the membrane thickness of 0.267 mm. Figure 4b and Figure 4c reveal the FESEM image at 1000× magnification, taken from top view of the membrane, and EDS result for neat PVDF membrane, respectively. From here, the fiber diameter was measured using ImageJ software (Windows version, Java 6) and was found to be at 2.471 µm. The EDS results reveal the composition of the neat PVDF membrane composing of primarily carbon, C and fluorine, F.

**Figure 5:**
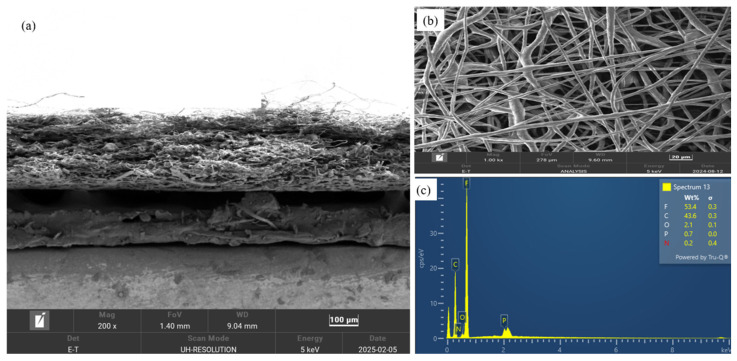
(**a**) FESEM image of membrane cross-section at 200× magnification, (**b**) FESEM image at 1000× magnification, and (**c**) EDS result for impregnated PVDF membrane with DEPHA/kerosene.

Figure 5 shows the FESEM image of impregnated PVDF membrane with DEPHA/kerosene. The cross-section image of the impregnated PVDF membrane denotes no significant changes on the membrane thickness from the neat PVDF membrane. However, the fiber diameter was increased from 2.471 µm to 2.958 µm, indicating membrane swelling. It should be noted that FESEM analysis was performed under high-vacuum conditions, and the sample preparation was conducted by trained technical personnel following standard procedures for membrane characterization, including removal of excess surface liquid prior to imaging. Due to the volatile nature of kerosene, partial evaporation during sample preparation and FESEM observation is expected. Therefore, the FESEM images should be interpreted as representing the morphological changes of the membrane after impregnation rather than direct visualization of the liquid phase. Nevertheless, the increase in fiber diameter suggests that a portion of the impregnating DEPHA/kerosene remained within the membrane structure, likely due to capillary forces and interactions with the PVDF matrix [24].

The EDS results reveal a change in the composition of the PVDF membrane, where the presence of phosphorous, P and oxygen, O was detected, albeit at low weight %. Note that the incorporation of DEPHA/kerosene into the PVDF membrane was through direct immersion and was done at a composition of 10–30 wt% DEPHA, with remaining percentage being kerosene, which explains the small percentage of P and O in the PVDF composition, despite possible partial loss of volatile components during the FESEM analysis process. From the FTIR results shown in Figure 3 and the FESEM-EDS results from Figure 5, it can be concluded that the PVDF membrane was successfully incorporated with DEPHA/kerosene. From here, the resultant membrane was used to evaluate the separation performance of Nd and Dy.

### 3.2. Separation of Nd and Dy

#### 3.2.1. The Effect of pH in Feed Solution

Different pH was used, from 0.7 and 1.85 to 5, to investigate the influence of pH of separating Nd and Dy. The range of pH was kept in acidic conditions due to the nature of extractant DEPHA, being an acidic extractant. The key mechanism of extraction is cation exchange, wherein the extractant DEPHA releases a H^+^ ion and exchanges it with the cation (REE^3+^). This reaction facilitates the movement or extraction of REE^3+^ into the organic phase. Once REE^3+^ enters the organic phase, the stripping solution exchanges the H^+^ ion to the extractant DEPHA, while REE^3+^ moves towards the stripping solution, thus regenerating the extractant for subsequent reaction [29,30,31]. Equation (4) describes the mechanism of cation exchange between REE^3+^ and DEPHA, where A is abbreviated for the extractant DEPHA [29]:

(4) $${REE}^{3+}+3HA\;\to REEA_{3}+3H^{+}\;\;$$

**Figure 6:**
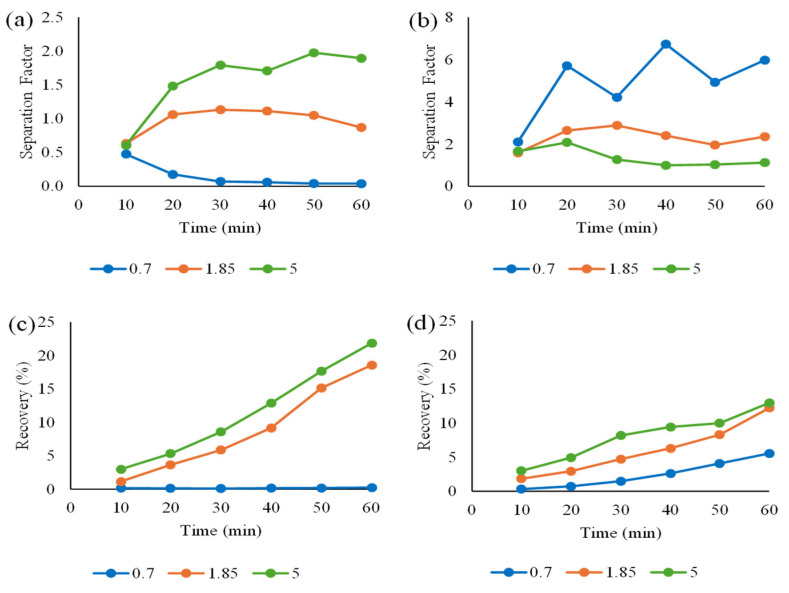
Separation factor of (**a**) Nd/Dy and (**b**) Dy/Nd, and recovery of (**c**) Nd and (**d**) Dy at different feed solution pH of 0.7, 1.85, and 5. Other conditions include DEPHA loading of 20 wt% and H_2_SO_4_ concentration of 3 M.

Figure 6 shows the separation performance between Nd and Dy. In terms of pH, the separation between Nd from Dy (Nd/Dy) favors a less acidic condition, notable at pH 5. Meanwhile, the separation of Dy from Nd (Dy/Nd) favors more acidic conditions, notably at pH 0.7. At low pH of 0.7, the separation factor of Nd/Dy showed a decreasing trend down to almost zero, whereas Dy/Nd showed an increasing trend up to 5.96 after 60 min. Conversely, at the higher pH of 5, the separation factor for Nd/Dy showed an upward trend till 1.89, while Dy/Nd showed a downward trend till 1.12 at 60 min. The results shown from this work reflect studies done elsewhere, where LREEs (in our case Nd) show better extraction at a higher pH [14,15] while HREEs (in our case Dy) favor lower pH [32].

In terms of recovery, when the pH of the feed solution was mildly acidic at 5, Nd was able to be recovered up to 21.82% while Dy was up to 12.97% after 60 min. Inversely, when the pH of the feed solution was more acidic at 0.7, the recovery of Nd was at its lowest, about 0.24%, while Dy was at 5.55% after 60 min. While pH does influence selectivity, it also affects the rate of transfer or recovery of REEs. Again, the observed relationship between separation factor, recovery, and pH is due to the nature of the DEPHA extractant and pH of the feed solution. A lower pH in the feed solution means the amount of H^+^ present in the feed solution is relatively high, while a higher pH in the feed solution means the amount of H^+^ present in the feed solution is relatively low. DEPHA, being an acidic extractant would be sensitive towards pH change as it affects the amount of H^+^ released by the extractant to facilitate REE^3+^ transport. In simpler terms, at lower pH in the feed solution, it would affect the rate of transfer of REE^3+^ since the presence of H^+^ ion is more on the feed solution and therefore reduces the rate of cation exchange, hence leading to lower REE^3+^ recovery [29].

In a nutshell, lower pH would have a higher selectivity of Dy/Nd but also lowers the rate of recovery. Comparatively, a higher pH would have a higher selectivity of Nd/Dy while also maximizing REE recovery. This work focuses on separating Nd from Dy; hence, a feed solution pH of 5 was selected for the next study.

#### 3.2.2. The Effect of Organic Carrier Loading

**Figure 7:**
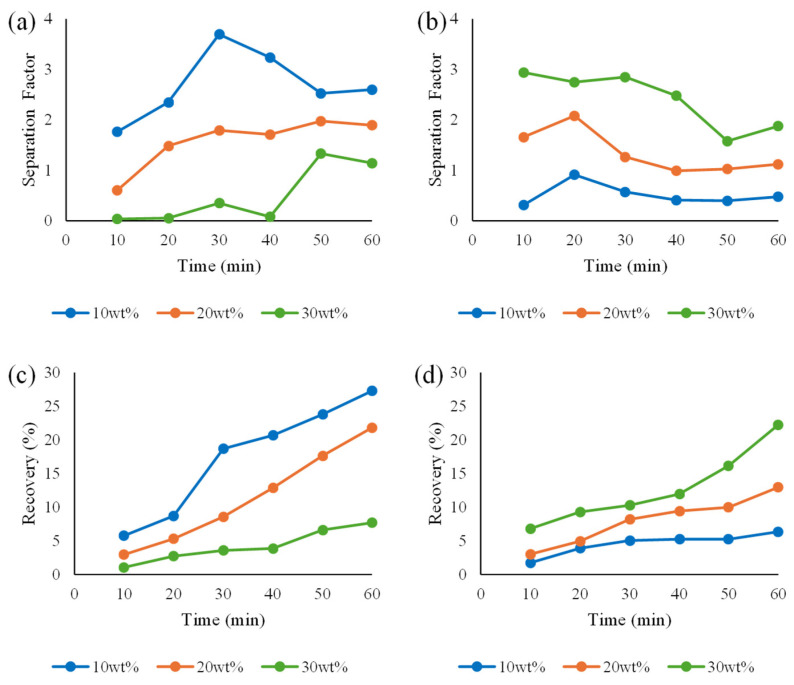
Separation factor of (**a**) Nd/Dy and (**b**) Dy/Nd, and recovery of (**c**) Nd and (**d**) Dy at different DEPHA loadings of 10 wt%, 20 wt%, and 30 wt%. Other conditions include feed solution pH of 5 and H_2_SO_4_ concentration of 3 M.

The organic carrier used for this study was an acidic extractant DEPHA and diluent kerosene. As the primary “carrier” to facilitate transport of REE^3+^ ions from the feed to the stripping solution, it is vital to investigate the role of organic carrier loading (wt% of DEPHA with respect to kerosene) in extraction and selectivity of Nd and Dy, especially when it is embedded in the polymer. Here, different DEPHA loading was used; 10 wt%, 20 wt%, and 30 wt%, with respect to wt% of kerosene. Figure 7 shows the separation performance between Nd and Dy.

In terms of separation factor, Nd/Dy prefers a lower DEPHA loading of 10 wt% while Dy/Nd favors a higher DEPHA loading of 30 wt%. This was primarily due to the ionic radii of Nd^3+^ and Dy^3+^ and its formation of a complex with DEPHA. In theory, the smaller the ionic radii, in this case Dy^3+^, the higher the stability of the complex that can be formed with the organic carrier, thus resulting in a higher distribution coefficient of Dy^3+^ from the aqueous feed to the organic phase [29,30,31]. This explained the selectivity of Dy/Nd with increasing DEPHA loading. Interestingly, for the separation of Nd, a maximum separation factor was achieved around 3.69 at about 30 min when using 10 wt% DEPHA. After 60 min, the separation factor of Nd/Dy for 10 wt%, 20 wt%, and 30 wt% DEPHA was 2.59, 1.89, 1.14, respectively. For Dy, the maximum separation factor was observed when using 30 wt% DEPHA at 10 min. After 60 min, the separation factor of Dy/Nd for 10 wt%, 20 wt%, and 30 wt% DEPHA was 1.87, 1.12, and 0.47, respectively. Similar findings from Gergoric, (2017) reported a higher separation factor between LREEs and HREEs can be established using a lower DEPHA loading, with minimal co-extractant of impurities [33].

The decline in separation factor may be due to (1) the system reaching saturation for a particular species and thus shifting towards the opposite species (in between the membrane and aqueous solution interface); (2) the availability or saturation of extractant in the pores of the membrane to facilitate cation exchange [12]; and/or (3) low stripping efficiency for the retained REE^3+^ in the polymer–organic carrier matrix. This was also evident in Figure 7c where at 10 wt% DEPHA, a steep slope of recovery was seen between 20–30 min, which correlates with the high separation factor in Figure 7a. Afterwards, the recovery of Nd was gradually increased up to 27.28% for 10 wt% DEPHA. For Dy, based on Figure 7d, a higher DEPHA loading of 30 wt% was preferred for maximizing the recovery of Dy by up to 22.23%, followed by 20 wt% DEPHA at 12.97% recovery and 10 wt% DEPHA at 6.36% recovery.

Apart from that, it is worth noting that the mechanism of cation exchange between DEPHA and REE^3+^ is highly related to the acidic properties of DEPHA and also the ionic radii and ionic charge of the REE. DEPHA is classified as an organophosphorus extractant, which is an acidic extractant. The principle of REE extraction involves DEPHA dissociating its H^+^ from the hydroxyl of the POO^−^ group and thereafter exchanging it with REE^3+^ at the anion of the POO^−^ [31]. Moreover, smaller ionic radii contribute to more stable complex formation with the extractant, resulting in higher distribution coefficients and consequently enhanced extraction of REEs. The order of highest extraction or distribution coefficient is also related to the atomic mass of the REE, i.e; La < Ce < Pr < Nd < Pm < Sm < Eu < Gd < Tb < Dy < Ho < Er < Tm < Yb < Lu [31,34]. From here, it shows that high separation factor of Dy/Nd can be achieved by using a higher DEPHA loading.

Overall, the organic carrier loading affects both the selectivity and recovery of Nd and Dy. For Nd, it is best to use a lower DEPHA loading compared to Dy. This result was also observed in another research by [35], where a high separation factor of Dy/Nd can be achieved by using a higher DEPHA loading. This work primarily focuses on Nd over Dy; thus, 10 wt% DEPHA loading was chosen for the next study on stripping solution concentration.

#### 3.2.3. The Effect of Stripping Concentration

The stripping solution used for this study was H_2_SO_4_ at various concentrations namely 1 M, 3 M, and 5 M. The concentration range and type of stripping solution was selected as reference from another study [15]. The function of a stripping agent was to free up loaded extractant, DEPHA from REE^3+^, thus regenerating the extractant for subsequent cation exchange (refer to Equation (4)) [29,30].

**Figure 8:**
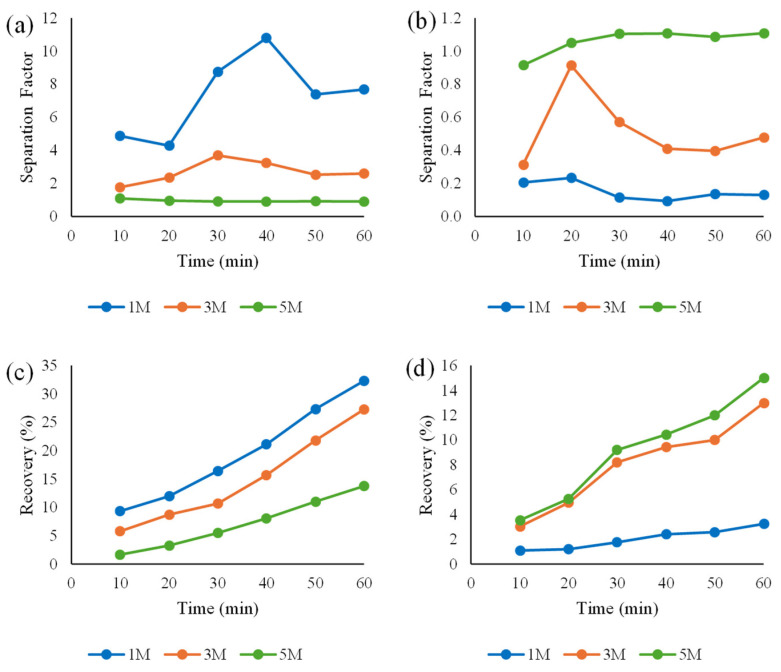
Separation factor of (**a**) Nd/Dy and (**b**) Dy/Nd, and recovery of (**c**) Nd and (**d**) Dy at different H_2_SO_4_ concentrations of 1 M, 3 M, and 5 M. Other conditions include feed solution pH of 5 and DEPHA loading of 10 wt%.

Figure 8a and Figure 8b shows the separation performance of Nd/Dy and Dy/Nd, respectively. For Nd/Dy, a higher separation factor was peaked at 10.78 in 40 min when using 1 M H_2_SO_4_ before gradually declining to 7.67 at 60 min. Then, 3 M showed the second highest separation factor at 2.59 and lastly 5 M at a steady 0.9 separation factor after 60 min. Comparatively, for Dy/Nd, the separation factor was relatively small for all three concentrations, with the best being at 1.11 separation factor when using 5 M H_2_SO_4_. In terms of recovery (Figure 8c,d), Nd showed a higher recovery when using 1 M H_2_SO_4_ up to 32.31%, followed by 3 M at 27.28% and 1 M at 13.77%. For Dy, 5 M H_2_SO_4_ showed the best recovery up to 15%, followed by 3 M at 12.97% and 1 M at 3.24%.

Supposedly, a higher concentration of stripping solution would be ideal because it would regenerate the extractant faster, hence improving rate of transport of REE^3+^ to the stripping solution. However, this was not the case, as seen from our results. The reason was due to the gradient of H^+^ in the system. A higher concentration of stripping solution contributes to higher amounts of H^+^ ions in the stripping solution. This causes an imbalance within the system, thus reducing REE^3+^ transport. Evidently, after 60 min, the pH of the feed solution was decreased from 5 to 0.9, similar to the stripping solution. What this means is that while REE^3+^ is being transported across the membrane from the feed solution, the higher gradient of H^+^ from the stripping solution was also transported to the feed solution, as a way for the system to reach equilibrium over the imbalanced H^+^ gradient. Similar findings were found from different studies where a lower molarity of stripping solution was preferred [12,15,36].

To summarize, whilst the concentration of H_2_SO_4_ does play a role in the selectivity of Nd and Dy, it does also influence the recovery rate. Ideally, a lower stripping concentration is preferred to ensure adequate transport of REE^3+^ from the feed to the stripping solution. In this case, 1 M H_2_SO_4_ was chosen together with 10 wt% DEPHA concentration for the next study on separation of a LREE and HREE from real PLS.

#### 3.2.4. Separation of Light and Heavy REEs

The experiment was conducted to determine the separation performance between light REEs (LREEs) and heavy REEs (HREEs). For the record, LREEs consist of lanthanum (La^3+^), cerium (Ce), praseodymium (Pr), and neodymium (Nd). HREEs consist of samarium (Sm), europium (Eu), gadolinium (Gd), terbium (Tb), dysprosium (Dy), holmium (Ho), erbium (Er), thulium (Tm), ytterbium (Yb), lutetium (Lu), scandium (Sc), and yttrium (Y). The PLS provided by our research partner, PRSB, consist of 43.62% LREE, 28.32% HREE, and 28.06% impurities (other heavy metal present during leaching). The pH of the PLS was around 2.1–2.3, however, and was not adjusted according to the findings in Section 3.2.1 to ensure the evaluation of the real condition of the PLS and hence not altering its original composition. Other conditions include DEPHA loading of 10 wt% and H_2_SO_4_ concentration of 1 M.

**Figure 9:**
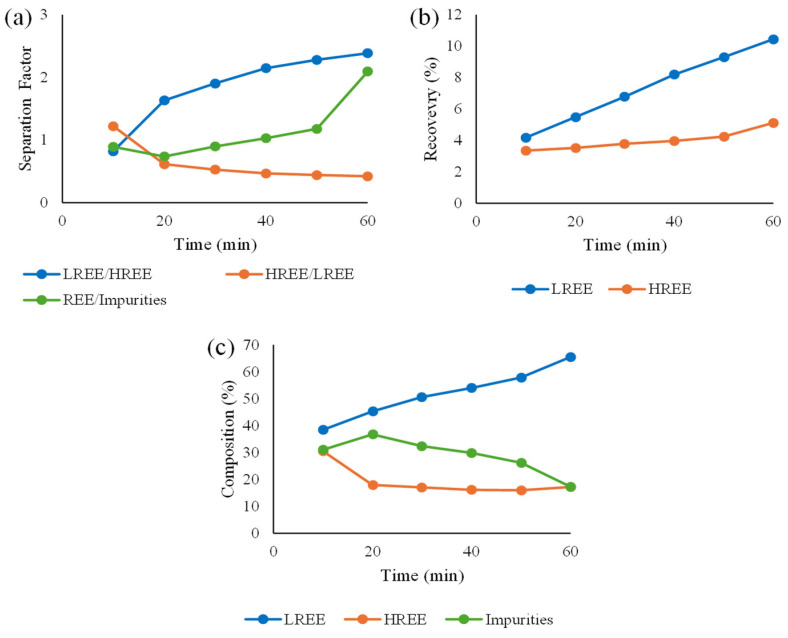
(**a**) Separation factor of LREE/HREE, HREE/LREE, and REE/impurities, (**b**) recovery of LREE and HREE, and (**c**) composition of LREE, HREE, and impurities in the stripping solution. Condition of experiment: DEPHA loading of 10 wt% and H_2_SO_4_ concentration of 1 M.

Figure 9a shows the separation performance when using real PLS. In the beginning, the separation factor of LREE/HREE is lower than HREE/LREE. After 20 min, the separation factor of LREE/HREE increases up to 2.38 at 60 min, signifying that the system is in favor of recovering LREE over HREE and impurities. It is also worth noting that with the presence of impurities such as thorium (Th), aluminium (Al), magnesium (Mg), potassium (K), calcium (Ca), iron (Fe), manganese (Mn), zinc (Zn), and lead (Pb) in the PLS, the system is more in favor of REEs, even after 60 min at 2.09 separation factor over total impurities.

In terms of recovery, more LREE is being stripped and recovered compared to HREE, as depicted in Figure 9b. At the beginning of the operation, the system was favoring HREE over LREE. After 20 min, the system shifted in recovering more LREE by up to 10.43% at 60 min. Although the percentage recovery of REE is low, it can be increased further by extending the operation, as discussed in the latter section on long term operation. A study done by Makowka and Pospiech, (2019), showed that using a membrane with an effective area of 12.56 cm^2^ was able to recovery about 5% of La^3+^ with DEPHA as the organic carrier in 60 min and up to 38.5% in 480 min [37]. In comparison, this work utilizes a PVDF membrane with a relatively smaller effective area of 4 cm^2^ and a 60 min stripping duration.

Looking further into the composition in the stripping solution as illustrated in Figure 9c, LREE was dominant, signifying that the rate of transfer of LREE is higher compared to HREE and impurities. In fact, the high rate of transfer of LREE into the stripping solution suppresses the composition of HREE and impurities in the stripping solution, which is ideal for separation of LREE and HREE from PLS. In this work, it is shown that the composition of LREE is at 65.47%, while HREE is at 17.25% and impurities at 17.28% for the duration of 60 min. Looking into the trends in Figure 9a,c, it is hypothesized that the composition of impurities in the stripping solution will further decline beyond 60 min, as the system favors REE over impurities. A similar finding from Baba et al., (2011), suggested using a SLM embedded with an acid extractant preferentially extracts REEs over ion impurities [38], which confirms the selectivity of REEs over ion impurities. Further, Middleton et al., (2023) explored the effect of competing impurities ions (Fe^2+^, Fe^3+^, and Al^3+^) over REE recovery and suggested that increasing the pH does have a positive impact towards Nd selectivity and mass transfer rate, due to the existing pH gradient between the feed and the stripping solution [14]. This work utilizes the existing pH gradient from the provided PLS (pH: 2.1–2.3) and stripping solution (~pH: 1), which favors more complexation of REEs as depicted in Equation (4) (if the pH is low, denoted by the high amount of H^+^, the equilibrium will shift towards the left, while a high pH, denoted by the low amount of H^+^ will cause the equilibrium to shift towards the right, favoring more REE complexation) [29,30]. It is also worth noting that although Fe^3+^ concentration may impede the REE recovery [14], the low concentration of impurities found in the PLS (this work) caused the system to favor more REE than impurities. As a suggestion, should the Fe^3+^ is found to be higher in the PLS (i.e., >10 mmol/L of Fe^3+^ [14]), another separation method must be implemented prior to the SLM to remove the impurities. Further details on the PLS content can be found in the Supplementary Materials.

#### 3.2.5. Long Term Operation

**Figure 10:**
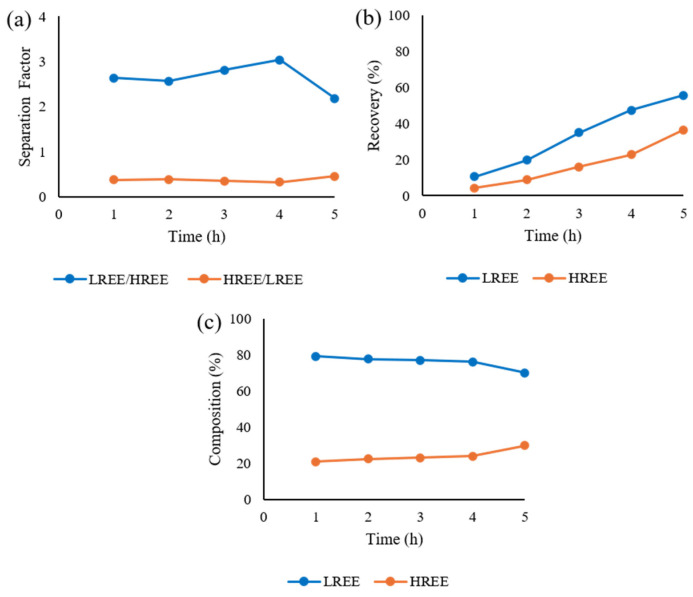
(**a**) Separation factor of LREE/HREE and HREE/LREE, (**b**) Recovery of LREE and HREE, and (**c**) composition of LREE and HREE in the stripping solution. Condition of experiment: DEPHA loading of 10 wt% and H_2_SO_4_ concentration of 1 M for 5 h.

The long-term stability study demonstrated that the SLM operated under the optimized conditions (10 wt% DEPHA, 1 M H_2_SO_4_) for 5 h of continuous operation. As previously stated, the impurities contents were not included in the long term study as the trend of the system were favoring more REE than impurities (as depicted in Figure 9). As illustrated in Figure 10a, the first 4 h of operation showed the separation factor for LREE/HREE remained relatively stable between 2.63–3.03 for 4 h, indicating that the membrane retained its preferential transport towards the LREE. Conversely, the HREE/LREE separation factor remained relatively constant between 0.32 and 0.38, further confirming that the selectivity of the membrane was largely unchanged during the initial stage of operation. This stable separation performance was reflected in the recovery profiles shown in Figure 10b, where LREE recovery reached approximately 47.31%, while HREE recovery remained considerably lower at 22.8% after 4 h. In addition, the composition of the stripping solution (Figure 10c) showed a consistently higher proportion of LREE than HREE, demonstrating that the carrier-mediated transport mechanism remained effective throughout the first 4 h without significant deterioration in membrane performance. However, after 4 h the separation performance began to change. At 5 h, the LREE/HREE separation factor decreased from approximately 3.03 to 2.17, while the HREE/LREE separation factor increased from 0.32 to 0.45. Simultaneously, HREE recovery increased markedly from 22.8% to 36.45%, whereas LREE recovery increased only modestly from 47.31% to 55.54% (a mere increment of 8.23%). The composition of the stripping solution also reflected this shift, with the LREE fraction gradually decreasing while the HREE fraction increased. These observations indicate that although the membrane remained functional throughout the 5 h experiment, its selectivity gradually shifted from favoring LREE towards a more balanced transport of both rare earth groups.

The observed decline in LREE selectivity after 4 h can be explained primarily by the development of concentration polarization across the membrane. As transport proceeded, LREE was progressively depleted from the feed solution while simultaneously accumulating in the stripping solution. At the end of t = 4 h, the feed solution contained approximately 42.58 ppm LREE compared with 40.49 ppm HREE, indicating that the concentration difference between the REE group had become considerably smaller overtime. Simultaneously, the stripping solution had become enriched with approximately 76.17% LREE in terms of composition. The reduced concentration gradient for LREE had diminished its driving force, whereas HREE still retained a relatively larger concentration gradient across the membrane. Consequently, HREE transport became increasingly favorable, leading to a relatively higher HREE recovery and a reduction in the overall LREE/HREE separation factor at t = 5 h. Apart from concentration polarization, membrane instability associated with partial loss of immobilization of organic carrier could have caused the decline in performance. A study conducted by Alemrajabi, (2022), reported that prolonged operation of SLMs exhibits gradual performance deterioration due to loss of organic extractant within the membrane pores, thereafter causing membrane instability [39]. Consequently, the combined effects of concentration polarization and progressive membrane instability provide a plausible explanation for the shift in selectivity observed after 5 h of continuous operation.

## 4. Conclusions

In conclusion, the SLM prepared by using electrospinning method showed viable results for the separation of Nd and Dy. This study was carried out to investigate the effect of different pH of the feed solution, organic carrier loading, and stripping concentrations. Ideally, to separate Nd from Dy, a higher feed solution pH of 5, a low loading of organic carrier at about 10 wt% and a low stripping concentration of 1 M H_2_SO_4_ was preferred. From there, the study was continued with the aforementioned conditions for the separation of LREE and HREE from real PLS. Here, a separation factor of 2.38 between LREE and HREE, and a recovery of 10.43% of LREE was achieved in under 60 min by using a small-scale membrane of 4 cm^2^ effective area. Long term operation suggested that the performance was favorable towards separation LREE/HREE in the first 4 h before shifting towards HREE, due to the combined effects of concentration polarization and membrane instability.

## Figures and Tables

**Table 1:** Parameters for electrospinning of PVDF solution.

Electrospinning Parameters	Value
Flow rate (mL/hr)	0.5
Applied voltage (kV)	15
Distance from the syringe tip to the collector (cm)	15

**Table 2:** Experimental parameters for separation of Nd and Dy.

Experimental Parameters	Value
pH of Feed Solution	0.7, 1.85, 5
Organic Carrier Loading (wt%)	10, 20, 30
Stripping Concentration (M)	1, 3, 5

## Data Availability

The data presented in this study are available on request from the corresponding author.

## Supplementary Materials

The following supporting information can be downloaded at https://www.mdpi.com/article/10.3390/polym18141742/s1, File S1: SLM Experiment using Real PLS (provided by PETRONAS Research using proprietary in-house leaching agent) under optimized condition (DEPHA 10wt% and 1M H_2_SO_4_ stripping solution) for 60 min.
