# Supplementary material for: Separation of Light and Heavy Rare Earth Elements via Electrospun Supported Liquid Membrane
Source: Polymers (Basel). 2026 Jul 16;18(14):1742. doi: 10.3390/polym18141742 (PMC13431360; doi:10.3390/polym18141742)
Supplement: Supplementary file 1 [file polymers-18-01742-s001.zip › polymers-4367918-supplementary.pdf]

Supplementary Data

Data for Figure 9: SLM Experiment using Real PLS (provided by PETRONAS Research using proprietary in-house leaching agent) under optimized condition (DEPHA 10wt% and 1M H_2SO_4 stripping solution) for 60 minutes.

		PLS Feed		Stripping Solution					
	Element	Initial t=0 min	60 min	10 min	20 min	30 min	40 min	50 min	60 min
Rare Earth Elements	Sc	0.04	0.04	0.00	0.00	0.00	0.00	0.00	0.00
	Y	22.74	22.19	0.52	0.55	0.55	0.55	0.54	0.55
	La	33.47	30.11	1.48	1.80	2.20	2.66	2.99	3.36
	Ce	7.25	6.46	0.32	0.44	0.53	0.64	0.71	0.79
	Pr	8.77	7.76	0.42	0.56	0.68	0.81	0.91	1.01
	Nd	31.33	28.06	1.15	1.63	2.06	2.52	2.90	3.26
	Sm	7.15	6.61	0.21	0.26	0.32	0.40	0.47	0.54
	Eu	0.34	0.32	0.01	0.01	0.01	0.02	0.02	0.02
	Gd	6.52	6.17	0.18	0.21	0.23	0.27	0.31	0.36
	Tb	1.18	1.05	0.12	0.12	0.12	0.12	0.13	0.13
	Dy	5.82	5.63	0.17	0.17	0.17	0.18	0.18	0.19
	Ho	1.21	1.09	0.13	0.13	0.13	0.13	0.13	0.13
	Er	3.30	3.21	0.08	0.09	0.09	0.09	0.09	0.09
	Tm	0.59	0.47	1.15	0.12	0.12	0.11	0.11	0.11
	Yb	3.08	3.04	0.05	0.05	0.05	0.05	0.04	0.04
	Lu	0.48	0.43	0.06	0.06	0.06	0.05	0.05	0.05
Impurities	Th	1.20	1.17	0.04	0.04	0.04	0.03	0.01	0.03
	Mg	1.69	1.69	0.00	0.00	0.00	0.00	0.00	0.00
	Al	14.67	13.85	0.98	1.05	0.96	1.13	0.94	0.82
	K	26.29	25.77	0.48	0.50	0.43	0.41	0.54	0.52
	Ca	1.79	1.22	0.60	1.33	1.75	1.45	1.03	0.57
	Mn	0.97	0.97	0.00	0.00	0.00	0.00	0.00	0.00
	Fe	1.48	1.43	0.57	0.56	0.20	0.43	0.64	0.05
	Cu	0.00	0.00	0.00	0.00	0.00	0.00	0.00	0.00
	Zn	0.24	0.22	0.06	0.07	0.05	0.07	0.09	0.02
	Pb	3.62	3.41	0.00	0.04	0.08	0.13	0.16	0.20
Conc. (ppm)	TREE	133.28	122.63	6.05	6.18	7.32	8.60	9.59	10.65
	LREE	80.82	72.39	3.37	4.43	5.48	6.62	7.51	8.43
	HREE	52.46	50.24	2.68	1.75	1.84	1.98	2.08	2.22
	Impurities	51.96	49.73	2.72	3.59	3.50	3.66	3.40	2.23
